# Research on Collaborative Delivery Path Planning for Trucks and Drones in Parcel Delivery

**DOI:** 10.3390/s25103087

**Published:** 2025-05-13

**Authors:** Ting Fu, Sheng Li, Zhi Li

**Affiliations:** College of Mathematics and Computer, Guangdong Ocean University, Zhanjiang 524088, China

**Keywords:** e-commerce platform, logistics delivery, truck–drone collaborative delivery, route optimization

## Abstract

With the rapid development of e-commerce, the logistics industry faces multiple challenges, including high delivery costs, long delivery times, and a shortage of delivery personnel. Truck–drone collaborative delivery combines the high load capacity of trucks with the flexibility and speed of drones, offering an innovative and practical solution. This paper proposes the Truck–Drone Collaborative Delivery Routing Problem (TDCRPTW) and develops a multi-objective optimization model that minimizes delivery costs and maximizes time reliability under capacity and time window constraints in multi-truck, multi-drone scenarios. To solve the model, an innovative two-stage solution strategy that combines the adaptive k-means++ clustering algorithm with temperature-controlled memory simulated annealing (TCMSA) is proposed. The experimental results demonstrate that the proposed model reduces delivery costs by 10% to 50% and reduces delivery time by 15% to 40%, showcasing the superiority of the truck–drone collaborative delivery model. Moreover, the proposed algorithm demonstrates outstanding performance and reliability across multiple dimensions. Therefore, the proposed approach provides an efficient solution to the truck–drone collaborative delivery problem and offers valuable insights for enhancing the efficiency and reliability of e-commerce logistics systems.

## 1. Introduction

The rapid economic growth in China has been influenced by the popularity and growth of e-commerce platforms in recent years. In 2023, China’s overall e-commerce revenue amounted to CNY 15.42 trillion, with rural e-commerce revenue totaling CNY 2.49 trillion. These factors have greatly contributed to enabling post-pandemic economic recovery in China. Despite significant development in e-commerce, it has encountered challenges including high logistical delivery costs, longer delivery times, and a lack of drivers for delivery. The rise of last-mile delivery has become a significant area of research as the logistics industry has advanced.

With technological improvements, unmanned aerial vehicles (UAVs), as an innovative technology [1], possess the benefits of rapidity, mobility, and cost-efficiency, and have proven their efficacy across multiple sectors, including aerial photography [2], agriculture [3], firefighting [4], medical services [5], and logistics [6]. Due to their unique advantages, UAVs possess considerable potential for research in the field of logistics and distribution. In 2013, Amazon in the United States initially proposed using drones for product delivery. By 2024, companies such as SF Express (SF Express Co., Ltd., Shenzhen, China), JD.com (JD Logistics, Beijing, China), and Meituan (Meituan Dianping, Beijing, China) had implemented standardized drone delivery routes successfully. In 2023, Zhang et al. [7] integrated the advantages of drones with urban logistics scenarios, establishing a logistics drone path planning model and conducting simulation experiments that demonstrated the benefits of using drones for logistics delivery. In 2024, Zhang et al. [8] conducted material research on drones, improving their wings to significantly reduce delivery costs in logistics applications. Chen et al. [9] designed a drone-based parcel pickup and delivery system based on a Radio Map, ensuring reliable communication quality during the parcel exchange process. In 2025, Liu et al. [10] proposed a two-stage algorithm for optimizing drone paths in logistics delivery, ensuring both energy efficiency and flight safety. These studies collectively validate the feasibility of drone technology in logistics delivery and highlight its growing maturity and potential in the field. Nevertheless, the smaller sizes of drones and their aptitude for short-range and smaller-scale deliveries impose constraints on servicing consumers with elevated demand at extended distances, whereas vehicles have superior load capacity and long-distance transport capabilities. Combining vehicles and drones for delivery could maximize efficiency.

Murray and Chu [11] introduced the concept of the Traveling Salesman Problem with Drone (TSP-D) in 2015, involving the collaborative delivery of goods by trucks and drones. References [12,13] focus on the truck–drone pickup and delivery problem to minimize economic costs. Reference [14] study the problem of truck–drone collaborative delivery to reduce delivery times. Gu et al. [15] proposed an innovative real-time method for truck–drone collaborative delivery. In this strategy, drones take off from specific truck docking points, complete package deliveries, and then return to the truck. R.J. Kuo [16] utilized trucks as warehouses, launching drones from designated locations and establishing a mixed-integer programming model aimed at minimizing costs. Bai et al. [17] proposed a planning model aimed at minimizing delivery time, allowing drones to carry multiple packages. In their research on a single-truck, multi-drone scenario, Chen et al. [18] studied a scenario with one truck and multiple drones, proposing a vehicle routing problem with time windows as constraints. Karaköse et al. [19] aimed to minimize delivery time and proposed a genetic algorithm-based method to solve the hybrid truck–multi-drone problem. In their research on the multi-truck, multi-drone scenario, Yin et al. [20] proposed considering the truck-based drone delivery routing problem with time windows. Luo et al. [21] established a mathematical model with the objective of minimizing cost and solved the one-to-one pickup and delivery problem with multiple trucks and multi-visit drones (OPDP-MTMV).

Existing related research primarily focuses on modeling and solving problems of minimizing delivery time or cost or optimizing delivery cost under time window constraints. While these studies propose various heuristic and exact algorithms to improve logistics efficiency and reduce operational costs, most models tend to focus on a single optimization objective or simplify multi-objective problems into weighted sum forms, failing to effectively reflect the complex trade-off between “service quality” and “cost control” that companies face. As a result, they do not comprehensively consider the dual demands of customer satisfaction and operational efficiency. Therefore, further exploration of modeling methods that integrate multi-objective optimization with practical operational needs is necessary to better balance customer demands and corporate benefits. For logistics companies, maintaining service quality while reducing delivery costs is key to promoting their development. For customers, shorter delivery times are crucial for improving their satisfaction with the service. However, the delivery time and cost of parcel delivery are related inversely. To balance these demands, this paper proposes the objective of maximizing time reliability [22]. Compared to simply minimizing time, minimizing time reliability can enhance customer satisfaction while also benefiting logistics companies. Therefore, this paper investigates the Truck–Drone Collaborative Delivery Routing Problem with Time Windows (TDCRPTW). Based on this, a mathematical model is established to solve the TDCRPTW with the dual objectives of minimizing both cost and time reliability.

The vehicle routing problem (VRP) is an NP-hard problem. Since the TDCRPTW is an extension of the VRP, the TDCRPTW is also an NP-hard problem. To solve it, one study [23] introduced a novel collaborative Pareto ant colony optimization algorithm and validated it through comparison with the Non-dominated Sorting Genetic Algorithm II (NSGA-II). Recently, simulated annealing (SA) has been widely applied to solve various VRP variants [24,25], as well as multi-objective optimization problems [26,27]. The path planning problem for truck–drone collaborative delivery is also a variant of the VRP problem. The authors of [28] developed an exact solution approach based on a time-expanded network flow model and a greedy heuristic algorithm to solve the path planning problem for truck–drone collaboration. For the TDCRPTW, this paper designs a two-phase algorithm that integrates an adaptive k-means++ clustering algorithm with TCMSA. In fact, the main contributions of this paper are as follows:

1. A multi-objective optimization model is developed to solve the TDCRPTW, considering both capacity constraints and time window restrictions in the multi-truck, multi-drone scenario.
2. In the study of truck–drone collaborative delivery, the concept of time reliability is introduced by this paper for the first time, addressing the needs of both logistics companies and customers by minimizing time reliability and delivery costs.
3. A two-stage strategy that integrates the self-adaptive k-means++ and temperature-controlled memory simulated annealing (TCMSA) approaches is proposed to solve the established model.

The rest of this paper is organized as follows: Section 2 summarizes the materials and methods used in the study. Section 3 presents the results. Section 4 discusses the findings and their implications.

## 2. Materials and Methods

**Figure 1:**
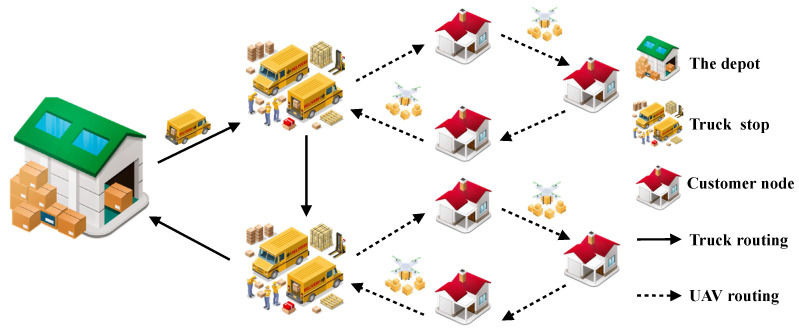
TDCDRPTW delivery process flowchart.

This paper focuses on a logistics network comprising multiple customer nodes, each serving only trucks and drones. The goal is to efficiently transport packages to client destinations promptly, reducing overall expenditure and enhancing punctuality within the constraints of accessible resources and customer requirements. Each truck transports the same quantity of drones to a central depot. Upon arriving at a predetermined truck stop, it sends the drones out to deliver the goods to nearby locations and then the drones autonomously return to the truck. Once all customer nodes have been served, the drones are returned to the depot. The delivery problem is denoted by TDCDRPTW, as shown in Figure 1.

### 2.1. Problem Assumptions

To facilitate permitting a comprehensive examination of the TDCDRPTW, the following assumptions are established:

1. Within the maximum load and mileage of the trucks and drones, the impact of travel speed on the travel range of trucks and drones is not considered.
2. The drone is considered to have completed a delivery upon arrival at the customer point, with unloading and receiving time not being considered at the customer node.
3. While conducting drone deliveries to customer nodes, the truck remains stationary at a truck stop.
4. Situations where delivery is hindered or delayed by causes beyond control are not taken into account.
5. The depot holds commodities that have consistent requirements.
6. Trucks and drones do not carry a load that exceeds their maximum load capacity.

### 2.2. Explanation of Parameters and Variables

The parameters and variables corresponding to the mathematical model of the TDCDRPTW are explained in Table 1 and Table 2.

In order to facilitate readers’ understanding, we have organized the commonly used abbreviations in this article, as shown in Table 3.

### 2.3. Objective

#### 2.3.1. Minimizing Total Economic Cost


(1) $$C_{f}=c\_truck\times \sum_{i\in U}\sum_{j\in U}x_{ij}^{f}d_{ij},$$

(2) $$C_{p}=c\_drone\times \sum_{i\in D}\sum_{j\in D}y_{ij}^{p}d_{ij},$$

(3) $$M_{1}=\sum_{k\in K}C_{f}+\sum_{k\in K}\sum_{i\in U}\sum_{j\in U}x_{ij}^{k}d_{ij}\alpha ,$$

(4) $$M_{2}=\sum_{s\in S}C_{p}+\sum_{s\in S}\sum_{i\in D}\sum_{j\in D}y_{ij}^{k}d_{ij}\beta ,$$

(5) $$\min M=M_{1}+M_{2}.$$

M represents the total economic cost, with $M_{1}$ being the cost for trucks and $M_{2}$ being the cost for drones. The costs of trucks include maintenance and logistics delivery expenses, while the costs of drones include maintenance and logistics delivery expenses. In this paper, the maintenance costs of trucks and drones are defined as the expenses incurred for maintenance and servicing during operation (such as oil changes, tire replacements, and filter changes) and the replacement of worn or faulty components and associated labor costs. Considering that in practical operations, maintenance costs are typically positively correlated with travel distance, they are modeled as cost functions linearly related to the distance traveled, as shown in Equations (1) and (2).

#### 2.3.2. Maximizing Time Reliability

**Figure 2:**
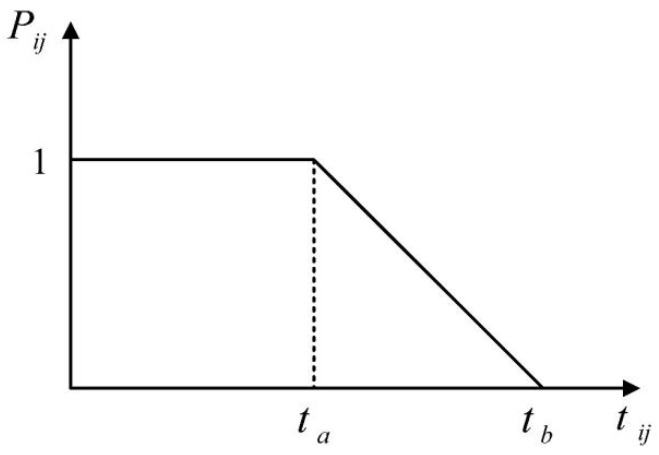
Time reliability function.

Generally, customers demand short delivery time. Thus, there is no minimum time requirement for delivery but there is a maximum time limit for delivery, which is a single-sided soft time window [29]. The time reliability function [30,31,32] has no impact on the objective function in the time range $\left[0,\;t_{a}\right]$. Considering that customer satisfaction generally decreases gradually over time within a reasonable delay range, this process is approximated as a linear function in this paper to simplify the computational complexity of the model. In the subsequent delay interval, the time reliability function reduces the value of the objective function at a particular rate in the time range $\left[t_{a},\;t_{b}\right]$. The time reliability function reduces the objective function to zero in the time range $\left[t_{b},\;\infty \right]$. The time reliability function is illustrated in Figure 2.

The objective function for maximizing time reliability is

(6) $$\max\;A_{T}=Truck\_P+Drone\_P,$$

Transform Equation (4) into a simpler form, as shown in Equation (5).

(7) $$\min-A_{T}=-(Truck\_P+Drone\_P),$$

where $A_{T}$ represents the total time reliability of trucks and drones, and

(8) $$Truck\_P=\sum_{k\in K}\frac{\sum_{i\in U}\sum_{j\in U}x_{ij}^{k}P_{ij}^{k}}{\sum_{i\in U}\sum_{j\in U}x_{ij}^{k}},$$

(9) $$Drone\_P=\sum_{s\in S}\frac{\sum_{i\in D}\sum_{j\in D}y_{ij}^{s}P_{ij}^{s}}{\sum_{i\in D}\sum_{j\in D}y_{ij}^{s}},$$

(10) $$P_{ij}=\left\{\begin{array}{l} 1,0\leq t_{ij}\leq t_{a},\\ \frac{t_{a}-t_{ij}}{t_{b}-t_{a}},t_{a}\leq t_{ij}\leq t_{b},\\ 0,t_{b}\leq t_{ij}. \end{array}\right.$$

(11) $$Truck\_t_{ij}=\frac{d_{ij}}{Truck\_v},$$

(12) $$Drone\_t_{ij}=\frac{d_{ij}}{Drone\_v}.$$

When calculating the time reliability of trucks, $t_{ij}=Truck\_t_{ij}$ and $t_{b}=Truck\_tb$; when calculating the time reliability of drones, $t_{ij}=Drone\_t_{ij}$ and $t_{b}=Drone\_tb$. Truck_P depicts the total time reliability of trucks, and Drone_P is the total time reliability of drones. $P_{ij}$ represents the time reliability from point i to point j, $P_{ij}^{k}$ is the time reliability of the k truck from point i to point j, and $P_{ij}^{s}$ is the time reliability of the s drone from point i to point j.

### 2.4. Model Constraints

The constraints of the mathematical model based on the TDCDRPTW are shown in Equations (13)–(24).

(13) $$\sum_{k\in K}Truck\_d_{k}\leq Q,$$

(14) $$\sum_{f\in S}z_{kf}Drone\_d_{s}\leq Truck\_d_{k},k\in K,$$

(15) $$\sum_{i\in U}demand_{i}\leq \sum_{k\in K}Truck\_d_{k},$$

(16) $$\sum_{j\in N}Point_{ij}\;demand_{j}\leq \sum_{f\in S}\sum_{j\in N}x_{in}^{k}z_{kf}R_{if}Drone\_d_{s},i,n\in U,k\in K,$$

(17) $$\sum_{i\in U}\sum_{j\in U}x_{ij}^{k}d_{ij}\leq Truck\_mileage,$$

(18) $$\sum_{i\in D}\sum_{j\in D}y_{ij}^{s}d_{ij}\leq Drone\_mileage,$$

(19) $$\sum_{k\in K}x_{ik}=1,i\in U,$$

(20) $$\sum_{s\in S}y_{is}=1,i\in N,$$

(21) $$\sum_{j\in U}x_{ij}^{k}=\sum_{j\in U}x_{ji}^{k},i\in U,$$

(22) $$\sum_{j\in D}y_{ij}^{s}=\sum_{j\in D}y_{ji}^{s},i\in D,$$

(23) $$\sum_{i\in D}\sum_{j\in D}Truck\_t_{ij}\leq Truck\_tb,$$

(24) $$\sum_{i\in D}\sum_{j\in D}Drone\_t_{ij}\leq Drone\_tb.$$

Constraint (13) specifies that the storage capacity at the depot must be equal to or greater than the total load capacity of the trucks. Constraint (14) specifies that the drone’s load at a truck stop must not exceed the load capacity of the corresponding truck. Constraint (15) states that the overall load capacity of the trucks must exceed or equal the total demand at customer nodes. Constraint (16) states that the total demand of customer nodes served at each truck stop must not exceed the total loading capacity of the drones on that truck. Constraints (17) and (18) limit the distance traveled by each truck and drone to within their maximum range. Constraint (19) states that there must be a one-to-one correspondence between each truck stop and the trucks that service it. Constraint (20) stipulates that each customer node is serviced by exactly one drone. Constraints (21) and (22) stipulate that whenever a truck or drone serves a truck stop or customer node, it is mandatory for it to enter and exit the node through the exact same point. Constraints (23) and (24) specify that each truck and drone must meet the latest time [33,34,35] limit for deliveries when distributing goods.

### 2.5. Optimization Algorithm

**Figure 3:**
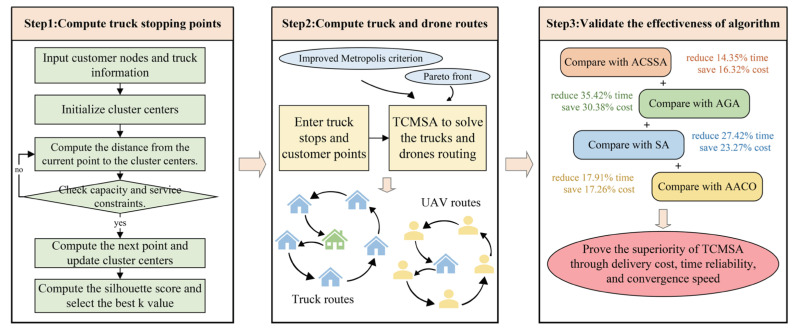
Algorithm’s structure and evaluation diagram.

Having established the model above, we will now focus on the algorithms to solve the model. This section introduces a two-stage optimization algorithm, used to solve the TDCDRPTW model previously formulated. In the first stage, an adaptive k-means++ algorithm is employed to determine the truck stop locations. In the second stage, the identified stop points and their cluster assignments are input into the TCMSA algorithm for collaborative route optimization between trucks and drones. To verify its effectiveness, the proposed approach is compared with traditional routing models: Adaptive Cooling Schedule Simulated Annealing (ACSSA), the Adaptive Genetic Algorithm (AGA), SA, and the Adaptive Ant Colony Optimization (AACO) algorithm. The detailed design and procedure of the two-stage algorithm will be elaborated in the subsequent sections. The algorithm’s structure (see Step 1 and Step 2) and evaluation (see Step 3) are shown in Figure 3.

#### 2.5.1. Truck Stop Location Strategy

Before solving the TDCDRPTW problem, it is necessary to determine the locations of the truck stops. The location of truck stops is intricately linked to the proximity and demand of customer nodes. Therefore, this paper uses k-means++ to select the truck stops. K-means++ requires the determination of the number of clusters k, depending on the dataset’s features and previous experience. However, the selection of k has an important effect on the clustering results. An unsuitable k value can directly influence the efficiency and effectiveness of the subsequent route planning. Therefore, this study improves k-means++ [36,37] by including an adaptive determination of k and constraints from Equations (14) and (16). This ensures the continuous fulfillment of drone delivery positions and improves the efficiency of the subsequent route planning.

The pseudo-code of the self-adaptive k-means++ algorithm is detailed in Algorithm 1.
**Algorithm 1** Pseudo-Code of Self-adaptive k-means++ Algorithm1***Input:*** Customer node, truck information, and parameters2***Output:*** Coordinates and capacity of each cluster and the cluster centers (truck stopping points) *%The output result is a cell array, where the first sublist of the cell array represents the truck docking points, and the remaining sublists correspond to the customer points contained within each truck docking point.*3Initialize cluster centers randomly from data. *%Adaptively set the value of k by searching for the best k within a certain range*.4***For*** each k in the given range of cluster numbers ***do***5        ***For*** each iteration in the range of Max number of iterations ***do***6               Compute distances from each data point to each cluster center.7               ***For*** each data point ***do***8                       Assign to the nearest cluster based on distance.                       *%Ensure that each category satisfies the capacity constraint.*9                       Check capacity constraint (14) and constraint (16)10                       ***If*** the assigned cluster exceeds capacity ***then***11                            Find the next best cluster for the assignment.12                       
***E***
***nd I***
***f***
13               
***E***
***nd F***
***or***
14               Update cluster centers based on assigned points15               Check for convergence based on the center change threshold.16               Calculate the silhouette score for the current k and save17       
***E***
***nd F***
***or***
18 
***E***
***nd F***
***or***
19  Determine the best k based on the maximum silhouette score.

In the self-adaptive k-means++ algorithm, the truck parking locations are ultimately determined by verifying whether the total customer demand within each truck parking cluster meets the constraint conditions and by evaluating whether the clustering performance is optimal. The distance formula, the calculation of the silhouette coefficient, and the formula for calculating new cluster centers used in the self-adaptive k-means++ algorithm are as follows:

(25) $$d(h,c_{i})=\sqrt{\sum_{j=1}^{n}{\left(h_{j}-c_{ij}\right)}^{2}},$$

(26) $$u_{i}=\frac{b_{i}-a_{i}}{\max(a_{i},b_{i})},$$

(27) $$c\prime_{i}=\frac{1}{\left|S_{i}\right|}\sum_{h\in S_{i}}h,$$

where $d\;(h,c_{i})$ expresses the distance matrix of each customer node to the cluster center, $u_{i}$ is the silhouette coefficient for the i cluster with a value of k, $c\prime_{i}$ represents the matrix of new cluster centers, and $S_{i}$ is the set of data points in the i cluster.

#### 2.5.2. Truck–Drone Collaborative Routing Optimization

The TDCDRPTW is a variant of the VRP [38]. Heuristic algorithms generally offer significant advantages in terms of computational speed, particularly for medium-to-large-scale problems. Among these, the SA method is prominent due to its efficiency and effectiveness especially. SA functions as a heuristic algorithm that incorporates stochastic elements to simulate the physical annealing process. Its core principle involves iteratively generating new candidate solutions from an initial solution, evaluating these options, and probabilistically accepting suboptimal solutions based on a predefined probability. As the “temperature” of the system decreases, the likelihood of accepting inferior solutions also diminishes, guiding the process toward a satisfactory optimization solution. However, traditional SA can encounter challenges such as extended computation times and difficulties in determining the best initial temperature T. To address these issues, this paper proposes TCMSA, which enhances the conventional SA approach by refining the initial temperature function, improving the Metropolis criterion, and introducing a new probability formula. Meanwhile, this paper also tracks the best solution at each temperature to prevent loss. The construction of the truck–drone path will be discussed below. The pseudo-code of the TCMSA algorithm is presented in Algorithm 2.
**Algorithm 2** Pseudo-Code of TCMSA Algorithm1:***Input*:** Clustering results, truck and drone delivery costs, algorithm parameters2:***Output*:** Best route, best cost, time, and time reliability3:***For*** f in the cell array composed of truck docking points and clustering results ***do***
        
*%After solving the previous clustering algorithm, the first sublist in the cell array represents the truck docking points, while the remaining sublists correspond to the customer points within each truck docking point. Therefore, the truck route should be solved when f = 1*
4:***If*** f stays in the truck docking points array (f = 1) ***then***

                
*%To facilitate computer recognition, trucks and drones are labeled, where flag = 0 represents solving the truck route, and flag = 1 represents solving the drone route.*
5:Optimize the truck route and mark flag = 0 6:Construct initial solution7:Decode the initial solution and calculate the initial cost
                  
*%Outer loop*
8:***For*** outIteriter in range of Max number of iterations ***do***
                           
*%Inner loop*
9:***For*** inIteriter in range of Max number of iterations ***do***
                
*%Ensure that the current solution satisfies the capacity constraint in the model.*
10:Generate neighborhood solutions under various constraints11:Decode the new solution12:Calculate the cost of the new solution13:***If*** the new solution’s objective compared to the current solution satisfies Pareto Best Solution ***then***14:Update the current solution15:***Else***
                           
*%Calculate the probability of accepting the new solution*
16:calculate the probability M of accepting the new solution17:***If*** random number <= M ***then***18:accept the new solution, update the current solution19:***End If***20:***End If***
                           
*%If the new solution satisfies the requirements of the Pareto frontier, update the current solution.*
21:***If*** the new solution’s objective compared to the Pareto frontier satisfies Pareto best Solution ***then***22:Update the current solution to the best Pareto frontier 23:***Else***24:***End If***25:***End For***26:update temperature27:record the best solution of each outer iteration28:***End For***29:***Else***30:optimize the drone route and mark flag = 131:follow the steps consistent with optimizing the truck route32:***End If***33:record the total global best solution for both truck and drone34:***End For***35:*%The above describes the process for solving the truck route. The process for solving the drone route is the same as that for the truck route.*

1. Setting Initial Parameters

To avoid losing sample information and due to the significant impact of the initial temperature on the solution, this paper improves the initial temperature setting [39,40,41]. Compared to traditional SA, assigning an initial temperature might result in failure directly. This study establishes the initial temperature by considering the particular features of the sample set. This approach ensures that all sample information is maximally utilized and the algorithm’s performance is optimized. The initial temperature and cooling function are as follows:

(28) $$T_{0}=-\frac{E(\max)-E(\min)}{\ln(e)},$$

(29) $$T_{k+1}=\omega \cdot T_{k},$$

where E(max) and E(min) represent the maximum and minimum function values corresponding to N randomly selected feasible solutions within the solution space, respectively. $T_{0}$ is the initial temperature, $T_{k}$ is the temperature after k cooling steps, ω is the cooling factor, and e∈[0.9,0.99] represents the probability of temperature adjustment, and in this study is set to 0.99.

2. Initial Feasible Solution

We generate $n+\sum_{k\in K}k-1$ initial feasible solutions from n customer nodes randomly. Additionally, we decode them to obtain the initial best solution, and set this initial solution as the current global best solution.

3. Generating New Solutions

We will provide a detailed explanation of the key steps involved in the generation of truck and drone routes using the example shown in the figure, focusing on the three key operations: Swap Structure, Reverse Structure, and Insertion Structure. Assume the node set is {0,1,2,3,4,5,11,12,13,14,0}, where Nodes 1, 2, 3, 4, and 5 are assigned to Truck 1. The route for Truck 1 is encoded as {0,1,2,3,4,5,0}.

- Swap Structure

**Figure 4:**
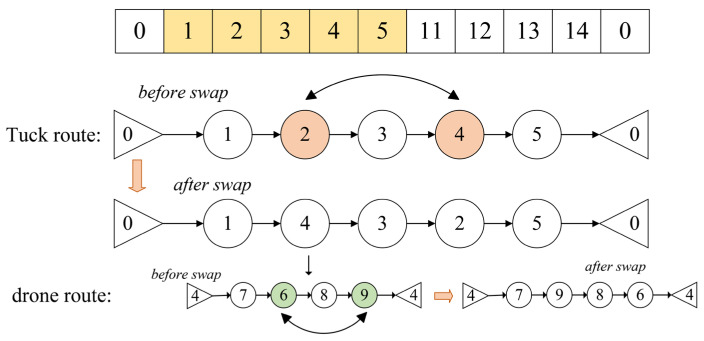
Swap Structure example diagram.

As shown in Figure 4, the Swap Structure refers to exchanging the positions of two nodes within the route. After performing a swap operation between Node 2 and Node 4, the new truck route becomes {0,1,4,3,2,5,0}. When the truck travels from Node 1 to 4, the drone departs from Node 4. The current drone route is encoded as {4,7,6,8,9,4}.After performing a swap operation between Node 6 and Node 9, the new drone route becomes {4,7,9,8,6,4}. This adjustment changes the visiting order of the nodes. The Swap Structure helps explore different node visiting sequences, thereby enhancing the diversity of the search process during optimization.

- Reverse Structure

**Figure 5:**
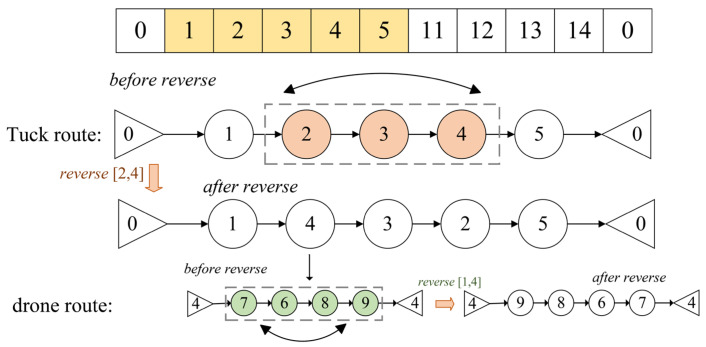
Reversal Structure example diagram.

As shown in Figure 5, the Reverse Structure refers to reversing the order of nodes within a specific segment of the route. Let [i,j] be the segment of nodes to be reversed for both the truck and the drone.

By reversing the segment [2,4] in the truck route, the new truck route becomes {0,1,4,3,2,5,0}. When the truck travels from Node 5 to Node 4, the drone departs from Node 4. The current drone route is encoded as {4,7,6,8,9,4}. After performing the operation on the segment between [1,4], the new route becomes {4,9,8,6,7,4}. This operation enables rapid adjustment of the local structure within the route, potentially improving the solution quality by reducing the route length or balancing the node load.

- Insertion Structure

**Figure 6:**
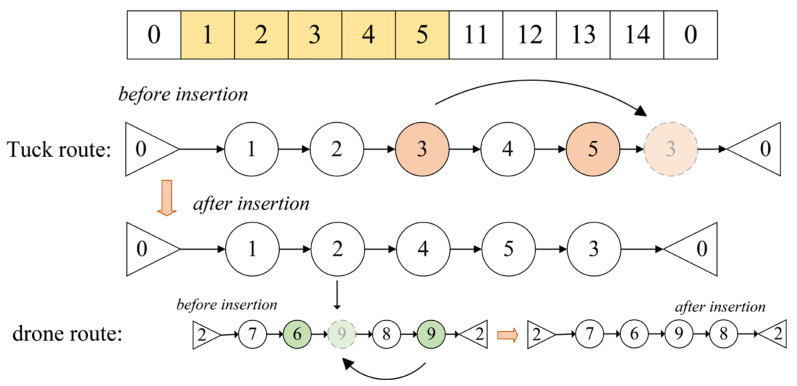
Insertion Structure example diagram.

As shown in Figure 6, the Insertion Structure refers to removing a node from its original position and inserting it into another position within the route. By removing Node 3 from its original position and inserting it after Node 5, the new truck route becomes {0,1,2,4,5,3,0}. When the truck travels from Node 1 to Node 2, the drone departs from Node 2. The current drone route is encoded as {2,7,6,8,9,2}. After removing Node 9 from its original position and inserting it after Node 6, the new drone route becomes {2,7,6,9,8,2}. This operation enhances the algorithm’s ability to fine-tune local searches by rearranging the visiting order of nodes, thereby optimizing the overall route structure.

4. Updating the Best Solution:

- Improved Metropolis criterion

In SA, two methods are employed to determine whether to accept new solutions, preventing the algorithm from being trapped in local optima. One method is to update the current solution to the best solution if the current solution is better. The second method is to accept a worse solution based on the Metropolis criterion with a certain probability.

To avoid instability in acceptance probability caused by the magnitude of differences between E(max)−E(min) and prevent the Metropolis criterion from overly relying on temperature adjustments and thus getting stuck in local optima, this paper improves the Metropolis criterion by introducing an influence factor S. This makes the probability of accepting worse solutions more reasonable. The improved Metropolis criterion formula is as follows:

(30) $$M_{3}=-e^{\frac{E(\max)-E(\min)}{S\cdot T_{k}}},$$

(31) $$S=-e^{\frac{E(\max)-E(\min)}{T_{0}}},$$

where $M_{3}$ represents the improved Metropolis criterion formula and S is calculated according to Equation (31).

After determining if the current solution is best and updating it, the algorithm will save the best solution from each iteration to avoid losing the true global best solution. The current solution is assessed using the cost and time reliability functions defined here.

- Pareto frontier

To improve the algorithm’s ability to assist business operators in selecting optimal solutions under various conditions, this paper selects solutions based on the Pareto frontier.

In constructing the Pareto frontier, if example x and y of a given instance satisfy Equations (32) and (33), and at least one objective is strictly better for x than y, then x dominates y and y does not belong to the Pareto frontier.

(32) $$f_{1}(x)\leq f_{1}(y),$$

(33) $$f_{2}(y)\leq f_{2}(x),$$

where $f_{1}(x)$ and $f_{1}(y)$ represent the time reliability of instances x and y, respectively, and $f_{2}(x)$ and $f_{2}(y)$ denote the delivery cost of instances x and y, respectively.

For the selection of solutions on the Pareto frontier, this paper introduces two weights to balance the requirements of the dual objectives, as shown in Equations (34) and (36).

(34) $$\max F(x)=\omega_{1}f^{1}(x)+\omega_{2}f^{2}(x),$$

(35) $$f^{1}(x)=\frac{f_{1}(x)-f_{1}^{\min}}{f_{1}^{\max}-f_{1}^{\min}},$$

(36) $$f^{2}(x)=\frac{f_{2}^{\max}-f_{2}(x)}{f_{2}^{\max}-f_{2}^{\min}},$$

where $\omega_{1}$ is the weight for time reliability and $\omega_{2}$ is the weight for delivery cost. $f^{1}(x)$ represents the normalized time reliability of instance x, $f^{2}(x)$ denotes the normalized delivery cost of the instance, $f_{2}^{\min}$ and $f_{2}^{\max}$ are the solutions with the lowest and highest delivery costs in the entire solution space, respectively, $f_{1}^{\min}$ and $f_{1}^{\max}$ are the solutions with the lowest and highest reliability in the entire solution space, respectively, and F(x) is a weighted objective function.

Business operators can set the weights based on actual conditions and requirements to select the most suitable solution. When business operators prioritize improving timeliness over reducing costs, $\omega_{1}>\omega_{2}$. Otherwise, $\omega_{1}<\omega_{2}$.Then, select the solution that achieves the max F(x).

In this paper, it is assumed that cost reduction is the optimal choice in the current scenario. Therefore, the solution with the lowest cost on the Pareto frontier is selected as the final result.

5. Termination

The algorithm terminates when the number of iterations reaches the set maximum value.

## 3. Results

### 3.1. Experimental Setup and Parameter Settings

1. Example Description

This paper selects the standard examples Set2a, Set2b, Set2c, and Set3 as the foundation for experiments provided by Breunig et al. [42]. To conform more closely to the model described in this work, the standard instances are modified in format, and the coordinate points and capacity sections are selected as the data used in this work. To ensure the accuracy and reliability of the research results, the solution algorithm and parameters used for truck–drone collaborative delivery are kept consistent with those used for single-truck delivery. Specifically, for the algorithm solving the single-truck delivery model, all steps in the TCMSA pseudo-code that involve drone path planning are the same as those in the truck–drone collaborative delivery model, except for steps 29–31.

2. k-means++ Parameter Settings

**Figure 7:**
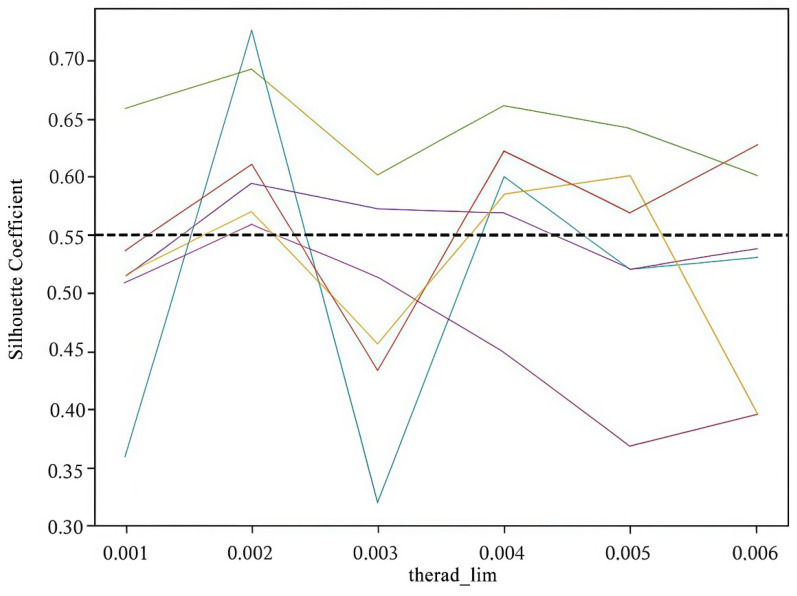
Clustering effect with different center threshold values.

The experiment involves setting the threshold value, threshold_lim, for the center change to a range of [0.001, 0.006] for experimentation. This paper shows the clustering effects within this particular range of values for several samples, as depicted in Figure 7.

From Figure 7, it can be observed that when therad_lim=0.002, the silhouette coefficients of the samples exceed 0.55, which is the highest compared to the other values. Therefore, this paper sets the center as therad_lim=0.002 in self-adaptive k-means++.

3. TCMSA Parameter Settings

Due to the large number of parameters in the TCMSA and the interdependencies among these parameters, this study utilizes the irace package [43] in R to determine the optimal parameter combinations. The final parameters identified are as follows: penalty coefficient = 20; maximum outer iteration = 1000; maximum inner iteration = 500; cooling factor = 0.99; and the probabilities for swapping, reversing, and inserting operations are set at 0.2, 0.4, and 0.4, respectively.

### 3.2. Model Performance Comparison

The TCMSA algorithm, tested using MATLAB R2023b on a computer with an AMD Ryzen 9 7950X 16-Core processor (Advanced Micro Devices, Santa Clara, CA, USA), 64 GB DDR5 memory, an RTX 4090D 24 GB graphics card (NVIDIA, Santa Clara, CA, USA), a 2 TB Acer SSD N7000 (Acer Inc., New Taipei City, Taiwan), and Windows 10 (Microsoft, Redmond, WA, USA), was used to solve the modified instances based on both the truck–drone collaborative delivery routing optimization model and the single-truck delivery routing optimization model. The results are presented in Table 4 and Table 5.

#### 3.2.1. Large-Scale Case Results

The results for large-scale examples with small payloads (50 customer nodes) are shown in Table 4. Table 4 demonstrates that the truck–drone collaborative delivery model produces a cost reduction ranging from 15% to 30% when compared to the single-truck delivery strategy. The largest saving observed in this model is 38%. The time cost is reduced by 30% to 50%, with a maximum reduction of 53% in delivery time. Additionally, the time reliability of the truck–drone collaborative delivery is generally better than that of the single-truck delivery.

#### 3.2.2. Medium- and Small-Scale Case Results

The results for the medium-scale (32 customer nodes) and small-scale (21 customer nodes) examples are shown in Table 5. Table 4 demonstrates that the truck–drone collaborative delivery model delivers a cost reduction ranging from 5% to 15% compared to the single-truck delivery approach. The maximum saving achieved by this collaborative model is 18%. The time cost is decreased by 15% to 40%, with a maximum decrease of 47% in delivery time. Additionally, the time reliability of the truck–drone collaborative delivery method is significantly better than that of the single-truck delivery method.

Overall, for small-scale, medium-scale, and large-scale examples, the truck–drone collaborative delivery model is more effective than the single-truck delivery strategy in terms of cost, delivery time, and time reliability. Truck–drone collaborative delivery has been shown to greatly save costs and delivery time, while also enhancing time reliability. The efficacy and indispensability of drones in the TDCDRPTW problem are apparent. Additionally, when comparing large-scale deliveries to medium- and small-scale deliveries, the model shows a higher percentage of cost savings and a decrease in delivery time. Thus, the model also overcomes the constraints of truck–drone collaborative delivery models that are only efficient in small-scale situations.

### 3.3. Algorithm Performance Comparison

#### 3.3.1. Clustering Performance

The silhouette coefficient, Davies–Bouldin Index (DBI), and Calinski–Harabasz Index (CH) are key metrics used to evaluate the quality of clustering. Their values range from [−1, 1], where a higher score indicates that samples within the same cluster are grouped more closely, and samples from different clusters are farther apart. The larger the silhouette coefficient, the better the clustering performance. A silhouette coefficient above 0.5 indicates good clustering. The DBI measures the ratio of the sum of intra-cluster distances to inter-cluster distances. The lower the DBI, the better the clustering performance. A DBI below 1 indicates good clustering and below 0.5 indicates excellent clustering. The CH index values clustering quality by measuring the ratio of between-cluster dispersion to within-cluster dispersion. The larger the CH index, the better the clustering performance. A CH value above 100 indicates good clustering and above 500 indicates excellent clustering.

**Figure 8:**
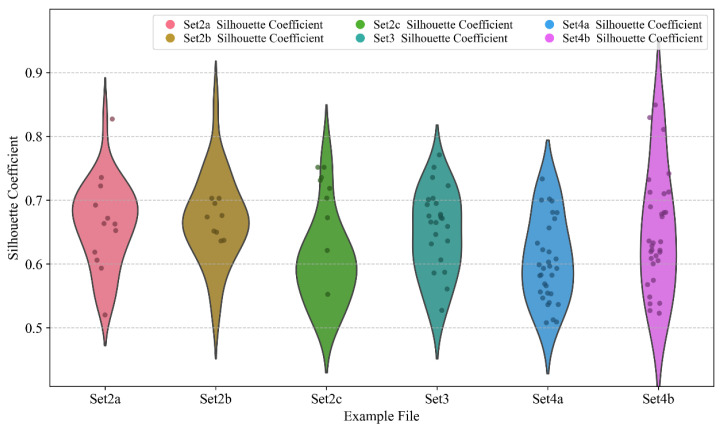
The silhouette coefficients of all examples.

**Figure 9:**
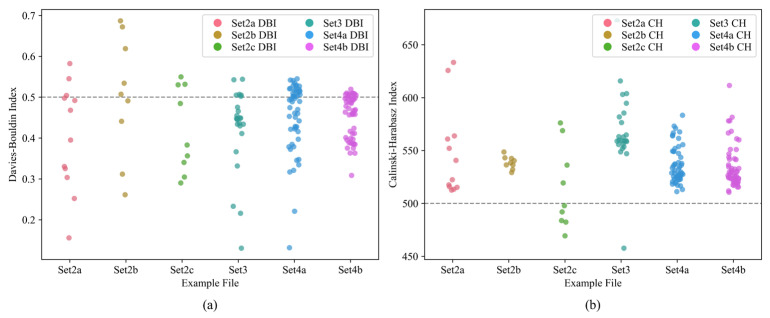
The DBI and CH index of all examples. (**a**) Davies–Bouldin Index of all examples; (**b**) Calinski–Harabasz Index of all examples.

The silhouette coefficient, DBI, and CH index for all examples are calculated, as shown in Figure 8 and Figure 9.

As shown in Figure 8 and Figure 9, the silhouette coefficients exceed 0.5 with fluctuations around 0.6 for all examples. Several examples even exceed a silhouette coefficient of 0.8. The DBI values are all below 1, with most of them being less than 0.5. The CH values are all above 100, with the majority exceeding 500. This indicates that the self-adaptive k-means++ algorithm we employed demonstrates a favorable performance.

#### 3.3.2. TCMSA Performance

To evaluate the effectiveness of the TCMSA approach employed in this study, we used TCMSA, ACSSA, SA, AGA, and AACO to solve the Set2b example, followed by a comparative analysis of their solution results. To ensure the reliability of the results, we used the irace package to select the best parameters for ACSSA, AGA, AACO, and SA. The conclusive results are shown in Table 6, Table 7 and Table 8.

**Figure 10:**
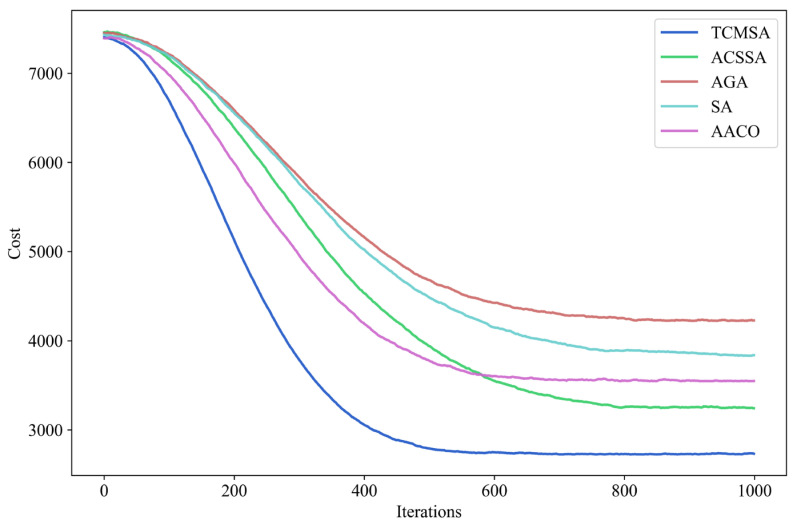
Comparison of iteration effects of different algorithms.

TCMSA achieves an average cost saving of 14.35% and an average reduction in delivery time of 16.32% compared to ACSSA. TCMSA achieves an average cost saving of 35.42% and an average reduction in delivery time of 30.38% compared to AGA. TCMSA achieves an average cost saving of 27.42% and an average reduction in delivery time of 23.27% compared to SA. TCMSA achieves an average cost saving of 17.91% and an average reduction in delivery time of 17.26% compared to AACO. To further validate the reliability of the proposed algorithm, we selected case s11–19 from Set2b and visualized the iterative results obtained from TCMSA, ACSSA, AGA, AACO, and SA, as shown in Figure 10.

The objective function value, initial iteration speed, solution variation, and convergence rate of TCMSA outperform those of ACSSA, SA, AGA, and AACO. Therefore, TCMSA demonstrates superior solution quality.

In addition, to further validate the superiority of TCMSA, we recorded the solution time of each algorithm when solving the Set2b example, as shown in Table 9.

As shown in Table 9, TCMSA outperforms ACSSA, AGA, SA, and AACO in solution time, demonstrating its superiority in this aspect.

In summary, TCMSA outperforms ACSSA, AGA, SA, and AACO across multiple dimensions. It not only demonstrates significant advantages in solution time, but also surpasses the other algorithms in terms of cost savings and reduction in delivery time. TCMSA achieves higher solution quality and faster convergence during the problem-solving process. Furthermore, the improvements in the initial temperature function, the Metropolis criterion, and the integration of the Pareto front have been proven to be effective in enhancing TCMSA’s performance. Therefore, TCMSA, as an efficient solution algorithm, holds great potential for wide application.

## 4. Discussion

This paper aims to address the high costs associated with logistics and the extended duration of deliveries encountered by e-commerce platforms through the introduction of a novel problem called the TDCDRPTW. To address this problem, we developed a nonlinear programming model with the objectives of minimizing economic cost and maximizing time reliability, and designed a two-stage algorithm that combines self-adaptive k-means++ with TCMSA to solve this mathematical model.

This study’s empirical findings indicate that the collaborative optimization model for truck–drone delivery paths achieves cost reductions ranging from 10% to 50% compared to the single-truck delivery path planning model. Additionally, delivery time is reduced by 15% to 40% and the model exhibits superior time reliability compared to single-truck delivery. The model exhibits superior performance in large-scale examples. Regarding the algorithm, the adaptive k-means++ clustering algorithm was used to select truck docking points and its silhouette coefficient consistently exceeded 0.5, which fully demonstrates the algorithm’s effectiveness and reliability. In addition, when solving the Set2b example, TCMSA achieved cost reductions of 14.35%, 32.86%, 27.42%, and 17.91%, respectively, and reduced delivery times by 16.32%, 30.38%, 23.27%, and 17.26% compared to ACSSA, AGA, SA, and AACO. Moreover, TCMSA also clearly outperformed the other algorithms in terms of solution time; this further confirms the superiority of the two-stage algorithm developed in this paper.

In addition, in order to effectively integrate the proposed truck–drone collaborative delivery system into existing logistics frameworks, it is recommended that enterprise managers first conduct small-scale pilot projects before full-scale deployment and gradually assess the system’s synergistic performance with traditional delivery methods under actual operating conditions. Delivery strategies should be adjusted in a timely manner according to the specific needs of customers in different regions. For example, the model proposed in this paper is primarily aimed at improving delivery efficiency in urban areas; however, the optimization objectives should be redefined to place greater emphasis on controlling delivery distances and reducing costs for rural or remote regions. In addition, managers should conduct market research to understand the expectations of users in different regions regarding service quality and delivery modes and flexibly adjust the frequency and methods of delivery services to further enhance user experience and the system’s adaptability.

In summary, the truck–drone collaborative delivery route planning model and the two-stage algorithm that integrates adaptive k-means++ with TCMSA presented in this paper provide valuable insights for addressing e-commerce logistics challenges. It should be noted that the model did not take into account certain factors, such as actual road traffic conditions (e.g., congestion) or the impact of natural conditions like wind speed on drone delivery speed and time. We will incorporate these factors and adjust the model according to actual e-commerce logistics scenarios to develop an extended version of the TDCDRPTW in future research.

## Figures and Tables

**Table 1:** Sets and parameters for the TDCDRPTW.

Parameter	Description
K={1,2,3,…,a}	Set of trucks
S={1,2,3,…,m}	Set of drones
U={1,2,3,…,t}∪{0}	Set of truck stops (cluster centers)
D={1,2,3,…,r}	Set of customer nodes and truck stops
N	Set of customer nodes, N=D\U
$C_{f}$	Maintenance cost per truck
$C_{p}$	Maintenance cost per drone
c_truck	Maintenance cost per unit distance for a truck
c_drone	Maintenance cost per unit distance for a drone
α	Delivery cost per km for trucks
β	Delivery cost per km for drones
Q	The capacity of the distribution center
$Truck\_d_{k}$	Load capacity of truck k
$Drone\_d_{s}$	Load capacity of drone s
Truck_mileage	Maximum mileage of a truck
Drone_mileage	Maximum mileage of a drone
Truck_v	Speed of the truck
Drone_v	Speed of the drone
Truck_tb	Latest time limit for the truck
Drone_tb	Latest time limit for the drone
$d_{ij}$	Distance between point i and point j
Demand	Demand at a customer node

**Table 2:** Explanation of variables.

Variable	Description
$x_{ij}^{k}$	Binary variable $x_{ij}^{k}\in \{0,1\},x_{ij}^{k}=1$ if truck k delivers from point i to point j; otherwise, $x_{ij}^{k}=0$.
$y_{ij}^{s}$	Binary variable $y_{ij}^{s}\in \{0,1\},y_{ij}^{s}=1$ if drone s delivers from point i to point j; otherwise, $y_{ij}^{s}=0$.
$Po{\mathrm{int}}_{ij}$	Binary variable $Po{\mathrm{int}}_{ij}\in \{0,1\},Po{\mathrm{int}}_{ij}=1$ if truck stop i contains customer node j; otherwise, $Po{\mathrm{int}}_{ij}=0$.
$z_{kf}$	Binary variable $z_{kf}\in \{0,1\},z_{kf}=1$ if drone f belongs to truck k; otherwise, $z_{kf}=0$.
$R_{if}$	Binary variable $R_{if}\in \{0,1\},R_{if}=1$ if drone f takes off from truck stop; otherwise, $R_{if}=0$.
$Truck\_t_{ij}$	Travel time of truck from node i to j.
$Drone\_t_{ij}$	Travel time of drone from node i to j.

**Table 3:** Commonly used abbreviations.

Abbreviation	Full Name
TDCRPTW	Truck–Drone Collaborative Delivery Routing Problem
VRP	Vehicle Routing Problem
SA	Simulated Annealing
TCMSA	Temperature-Controlled Memory Simulated Annealing
ACSSA	Adaptive Cooling Schedule Simulated Annealing
AGA	Adaptive Genetic Algorithm
AACO	Adaptive Ant Colony Optimization
DBI	Davies–Bouldin Index
CSP	Cost Savings Ratio
PRT	Proportion of Time Savings

**Table 4:** Results of large-scale example solutions.

Example Name	Truck–Drone			Single Truck			CSP	PRT
	Cost/CNY	Time/h	RT	Cost/CNY	Time/h	RT		
Set2b-s11-19	2726.08	5.67	3.2	3779.83	9.25	2.7	0.28	0.39
Set2b-s11	2684.27	5.72	3.11	4457.34	8.29	1.72	0.4	0.31
Set2b-s2-17	2879.55	6.24	4.4	4808	10.37	1.18	0.4	0.4
Set2b-s2-4	2987.83	6.48	3.33	4466.95	9.57	4	0.33	0.32
Set2b-s27-47	2758.52	6.22	3.6	4941.84	8.42	1.39	0.44	0.26
Set2b-s32-37	2976.99	6.86	3.84	4359.72	8.06	3	0.32	0.15
Set2b-s4-46	2898.58	6.95	3.4	4086.58	9.09	3	0.29	0.24
Set2b-s6-12	4464.73	8.64	3.25	6245.1	13.83	3	0.29	0.38
Set2b-s6-12	4162.47	8.76	4	6146.73	15.39	3	0.32	0.43
Set2c-s11-19	3973.4	8.32	3.4	6454.57	17.48	1.13	0.38	0.52
Set2c-s11-19	4420.51	8.58	3.67	5697.71	15.21	3	0.22	0.44
Set2c-s2-17	4104.38	8.72	3.6	5349.4	15.8	1.95	0.23	0.45
Set2c-s2-4	4092.03	9.64	3.6	6170.32	16.2	4	0.34	0.4
Set2c-s27	4101.02	8.18	3.6	6312.69	17.23	−0.76	0.35	0.53
Set2c-s32-37	4226.18	8.48	4	5821.31	12.42	0.32	0.27	0.32
Set2c-s4-46	4142.72	8.38	3.8	5973.71	15.05	0.68	0.31	0.44
Set2c-s6-12	4154.87	8.84	3.33	6536.34	17.46	4	0.36	0.49
Set2c-s6-12	4127.78	9.17	4	6425.83	16.14	1.93	0.36	0.43
Set3-s12-18	4779.97	10.05	3.6	5987.39	14.32	3	0.2	0.3
Set3-s12-41	5040.04	9.34	2.71	6687.89	16.27	1.96	0.25	0.43
Set3-s12-43	5003.54	10.81	3.4	5471.38	14.47	3	0.09	0.25
Set3-s13-19	3885.16	8.77	3	5980.94	12.98	1.58	0.35	0.32
Set3-s13-42	4109.34	9.18	3.8	5789.92	17.62	3	0.29	0.48
Set3-s13-44	3847.67	7.65	3	5955.23	12.45	3	0.35	0.39
Set3-s39-41	4931.01	10.25	3.19	5988.51	14.9	−0.43	0.18	0.31
Set3-s40-41	5201.63	10.36	3.6	6120.67	13.45	2	0.15	0.23
Set3-s40-42	3991.61	9.53	3.8	5886.83	14.23	0.95	0.32	0.33
Set3-s40-43	4863.76	9.86	2.98	5771.63	15.02	1.95	0.16	0.34
Set3-s41-42	4278.31	8.58	3.53	6102.58	13.72	0.54	0.3	0.37
Set3-s41-44	4098.85	9.63	3.4	6284.25	14.54	1.31	0.35	0.34

Note: CSP and PRT refer to the proportion of cost savings and time; RT refers to time reliability.

**Table 5:** Results of small-scale and medium-scale example solutions.

Example Name	Truck–Drone			Single Truck			CSP	PRT
	Cost/CNY	Time/h	RT	Cost/CNY	Time/h	RT		
2a-s10-14	3623.74	7.4	2.1	4107.54	13.9	1.39	0.12	0.33
2a-s11-12	3516.86	7.09	3.2	4265.93	11.34	1	0.18	0.37
2a-s12-16	3752.4	9.1	3.6	3982.56	11.95	2	0.06	0.24
2a-s6-17	3880.48	10.42	3	4107.55	12.07	1	0.06	0.14
2a-s8-14	3682.54	8.82	3.8	3784.94	12.63	1.36	0.03	0.3
2a-s9-19	3694.42	7.81	3.4	4230.01	13.95	2	0.13	0.44
2a-s1-9	6599.59	14.19	3.69	6607.98	16.98	1	0	0.16
2a-s14-22	6369.18	15.12	2.49	6456.26	16.46	2	0.01	0.08
2a-s2-13	6191.38	13.93	2.66	6681.76	16.49	0.74	0.07	0.16
2a-s3-17	6578.02	16.8	2.64	6623.8	17.36	1	0.01	0.03
2a-s4-5	6449.46	15.91	2.83	6442.04	16.74	1	0	0.05
2a-s7-25	6456.05	15.62	2.6	6601.84	18.79	1	0.02	0.17
3_s13-14	4003.23	9.84	3	4173.15	12.21	2	0.04	0.19
3-s13-16	3839.92	10.01	3.2	3884.69	13.61	1	0.01	0.26
3-s13-17	3827.42	9.79	2.98	3972.85	12.35	2	0.04	0.21
3-s14-19	4026.43	10.1	3.4	4142.74	12.97	1	0.03	0.22
3-s17-19	3999.97	8.98	3	4086.71	12.3	1.31	0.02	0.27
3-s19-21	4955.79	12.29	2.2	4946.08	12.98	1.11	0	0.05
3-s16-22	6090.11	13.05	3.33	6120.78	16.86	0	0.01	0.23
3-s16-24	6025.81	15.73	3.35	6068.89	17.57	2	0.01	0.1
3-s19-26	6155.29	13.03	2.26	6292.85	18.14	0.25	0.02	0.28
3-s22-26	6410.39	14.02	2.5	6414.05	18.2	1	0	0.23
3-s24-28	6078.47	15.24	2.84	6176.68	17.88	2	0.02	0.15
3-s25-28	6096.26	12.55	2.61	6282.15	17.1	1	0.03	0.27

**Table 6:** Results of different algorithms on the Set2b example file 1.

Example	TCMSA			ACSSA			AGA		
	Cost/CNY	Time/h	RT	Cost/CNY	Time/h	RT	Cost/CNY	Time/h	RT
s11-19	2726.08	5.67	4	3242.99	7.51	3.2	4223.86	9.21	2.8
s11	2684.27	5.72	3	3463.94	8.26	2.94	3952.55	8.05	3.32
s2-17	2879.55	6.24	3.75	3316.9	7.83	3	4528.54	8.78	2.97
s2-4	2987.83	6.48	3	3406.31	7.46	2.78	4226.19	9.54	2.54
s27-47	2758.52	6.22	3	3138.01	7.35	2.4	4932.23	8.43	2.78
s32-37	2976.99	6.86	2.8	3464.36	7.81	3.29	4487.17	9.95	2.5
s4-46	2898.58	6.95	2.63	3321.68	8.4	2.66	4859.48	9.75	2.65

**Table 7:** Results of different algorithms on the Set2b example file 2.

Example	SA			AACO		
	Cost/CNY	Time	RT	Cost/CNY	Time	RT
s11-19	3832.34	8.35	2.6	3548.08	7.53	3.25
s11	3442.3	7.16	3.25	3346.64	7.12	3
s2-17	4327.87	8.44	2.75	3387.2	7.25	2.56
s2-4	3513.7	7.58	2.85	3350.43	7.75	2.98
s27-47	4320.14	7.97	3.25	3364.33	7.48	2.71
s32-37	4380.14	9.54	2.87	3558.34	7.66	3.25
s4-46	4249.67	9.1	3	3952.6	8.68	2.56

**Table 8:** Comparison of solution effects of different algorithms.

T->ACSSA		T->AGA		T->SA		T->AACO	
CSP	PRT	CSP	PRT	CSP	PRT	CSP	PRT
0.16	0.25	0.35	0.38	0.29	0.32	0.23	0.25
0.23	0.31	0.32	0.29	0.22	0.2	0.2	0.2
0.13	0.2	0.36	0.29	0.33	0.26	0.15	0.14
0.12	0.13	0.29	0.32	0.15	0.15	0.11	0.16
0.12	0.15	0.44	0.26	0.36	0.22	0.18	0.17
0.14	0.06	0.34	0.31	0.32	0.28	0.16	0.12
0.13	0.17	0.40	0.29	0.32	0.24	0.27	0.2

**Table 9:** Solution times of different algorithms for Set2b.

Example	TCMSA	ACSSA	AGA	SA	AACO
s11-19	50.91s	59.25s	64.02s	55.83s	56.98s
s11	61.11s	67.23s	68.34s	66.92s	64.57s
s2-17	69.46s	75.42s	77.23s	74.91s	71.03s
s2-4	79.51s	86.37s	90.76s	85.11s	86.28s
s27-47	90.42s	98.53s	99.74s	97.11s	98.15s
s32-37	108.13s	110.36s	112.15s	109.72s	110.54s
s4-46	120.15s	141.87s	154.21s	135.88s	128.02s

## Data Availability

The data used in this study were sourced from the publicly available databases cited in Breunig et al.’s article [42]. These datasets are publicly archived and can be accessed through the links provided in the original article. For further information, please contact the corresponding author.
